# Impact of repeated four-monthly anthelmintic treatment on ***Plasmodium ***infection in preschool children: a double-blind placebo-controlled randomized trial

**DOI:** 10.1186/1471-2334-10-277

**Published:** 2010-09-21

**Authors:** Patrick Kirwan, Andrew L Jackson, Samuel O Asaolu, Sile F Molloy, Titilayo C Abiona, Marian C Bruce, Lisa Ranford-Cartwright, Sandra M O' Neill, Celia V Holland

**Affiliations:** 1 Department of Zoology, University of Dublin, Trinity College, Dublin 2, Ireland; 2 Department of Zoology, Obafemi Awolowo University, Ile-Ife, Nigeria; 3 HIV/AIDS Research and Policy Institute, Chicago State University, Chicago, IL, USA; 4 Faculty of Biomedical and Life Sciences, Parasitology, Glasgow Biomedical Research Centre, University of Glasgow, Glasgow G12 8QQ, UK; 5 School of Nursing, Dublin City University, Dublin 9, Ireland

## Abstract

**Background:**

Helminth infections can alter susceptibility to malaria. Studies need to determine whether or not deworming programs can impact on *Plasmodium *infections in preschool children.

**Methods:**

A double-blind placebo-controlled randomised trial was conducted to investigate the impact of anthelmintic treatment on *Plasmodium *infection in children aged 12-59 months. Children were randomly assigned to receive either albendazole or placebo every four months for 12 months with a follow-up at 14 months.

**Results:**

320 children (out of 1228, 26.1%) complied with all the follow-up assessments. *Plasmodium *prevalence and mean *Plasmodium *parasite density was significantly higher in the treatment group (44.9% and 2319 ± SE 511) compared to the placebo group (33.3% and 1471 ± 341) at baseline. The odds of having *Plasmodium *infection increased over time for children in both the placebo and treatment groups, however this increase was significantly slower for children in the treatment group (P = 0.002). By month 14, mean *Plasmodium *density had increased by 156% in the placebo group and 98% in the treatment group but the rate of change in *Plasmodium *density was not significantly different between the groups. The change from baseline in haemoglobin had a steeper increase among children in the treatment group when compared to the placebo group but this was not statistically significant.

**Conclusions:**

Repeated four-monthly anthelminthic treatments for 14 months resulted in a significantly lower increase in the prevalence of *Plasmodium *infection in preschool children which coincided with a reduction in both the prevalence and intensity of *A. lumbricoides *infections.

**Trial Registration:**

Current controlled trials ISRCTN44215995

## Background

Infections with multiple parasitic species are common in nature [1]. Synergistic and competitive interactions can occur between parasite species, which can influence the likelihood of their successful transmission to other hosts and increase or decrease their overall pathogenic impact [2]. It is estimated that over a third of the world's population are infected with helminths [3] or one or more of the *Plasmodium *species [4]. Populations infected with helminths and *Plasmodium *are mainly confined to the tropics and subtropics and this results in high rates of co-infection [2]. It has been demonstrated that helminth infections can impact on *Plasmodium *parasite densities [5] and alter susceptibility to clinical malaria[6]. Helminth-malaria interactions have been studied in animals models [7] and humans [5,8-11]. To date human studies investigating helminth-malaria interactions have been carried out in older age groups with contrasting results [12,13].

In 2001, the World Health Assembly passed a resolution urging member states to control the morbidity of soil transmitted helminth (STH) infections through large-scale use of anthelminthic drugs for school-aged children in less developed countries [14]. It has also been recommended that preschool children should be included in these deworming programs [15]. Given that preschool children, defined as aged less than five years, make up between 10%-20% of the two billion people worldwide who are infected with STHs [3] and young children are a high risk group for malaria [16], it is essential to determine whether or not deworming programs have the potential to significantly impact on *Plasmodium *infections in preschool children.

The need for well-designed longitudinal intervention studies to examine the relationship between helminths and malaria has been acknowledged [17]. To date, two intervention studies in the published literature have investigated the relationship between *A. lumbricoides *and malaria, both of which have included school age children and adults [5,6]. We therefore conducted the first double-blind placebo-controlled randomised trial to establish the effect of repeated four-monthly anthelminthic treatments for 14 months on the prevalence of Plasmodium infection and Plasmodium parasite density in preschool children living in four semi-urban communities in Nigeria where STH prevalence is high.

We have previously reported that 50% of the preschool children recruited into this trial were infected by one or more helminths, the most prevalent STH being *A. lumbricoides *(47.6%), and that repeated four-monthly treatments were successful in significantly reducing the prevalence and intensity of *A. lumbricoides *infections[18]. This paper investigates the potential impact of anthelminthic treatment on Plasmodium infection in the same cohort of children.

## Methods

### Study area and participants

The study was carried out between May 2006, and August 2007, in four semi-urban villages, Akinlalu, Ipetumodu, Moro and Edunabon, situated near Ile-Ife, Osun State, Nigeria. The dry season extends from November to March while the rainy season occurs from April to October [19]. STHs and malaria are endemic in this region [18,20]. Malaria transmission is intense, occurring all year round, with a major peak during the rainy season [20]. *P. falciparum *is the predominant species, present in the majority (99.5%) of Plasmodial infections, while *P. malariae *and *P. ovale *are present in 17% and 9.8% of infections respectively [21]. The study area, participants, enrolment, study design, randomization and sample size estimate have been previously described[18]. Informed consent was obtained from each mother to enroll their child. The study protocol was approved by the Ethics and Research Committee, Obafemi Awolowo University Teaching Hospitals' Complex, Ile-Ife, Nigeria.

### Study design

The study was a double-blind placebo-controlled randomised trial. Children aged 12-59 months were randomly assigned to receive either albendazole or placebo every four months for 12 months with a follow-up at 14 months. Children who complied with all the assessments received either treatment (albendazole) or placebo tablets on four occasions. Children aged one year received 200 mg of albendazole (1 tablet) and children aged ≥ 2 years received 400 mg (2 tablets) of albendazole [15]. Children in the placebo group were treated with albendazole at the end of the study. This trial is reported in accordance with the CONSORT guide-lines for randomised studies [22].

Children were screened for STHs and Plasmodium at baseline, 4, 8, 12, and 14 months. Stool samples were obtained before treatment was provided and stools were processed by formol-ether concentration [18]. To maintain consistency all stool samples (one per child per time point) were examined by PK. The primary outcomes were infection with *Plasmodium *spp., *Plasmodium *density, and malaria attacks. The secondary outcomes were haemoglobin concentration and nutritional status. Finger-prick blood samples were used to undertake a malaria rapid test and make thick and thin blood smears for each child. *Plasmodium *was diagnosed using Parascreen rapid diagnostic tests (RDTs; Zephyr Biomedicals, Verna Industrial Estate, Verna Goa, India). In line with local practice, on the day of the assessment, an 'uncomplicated' malaria attack was defined as a positive RDT and a fever (> 37.5°C).

Children suffering a malaria attack were treated with Coartem (artemether-lumefantrine) [23]. Data from Parascreen RDTs are not presented in this paper. For the purpose of analysis, malaria attacks were defined retrospectively as children with a fever (> 37.5°C) who were diagnosed with *Plasmodium *parasites by microscopy of blood slides. Due to the overwhelming prevalence of *P. falciparum *in this region, the infections were not further divided into those with *P. malariae *or other species for the analysis. Slides were stained with a 3% Giemsa solution [24] and examined using a × 1000 oil immersion magnification by the Kenya Medical Research Institute (KEMRI). Total *Plasmodium *density was measured by counting the number of asexual parasites against 200 leucocytes in the thick blood film and assuming an average of 8000 leucocytes/μL of blood.

Anthropometric measurements (height and weight) and nutritional assessments have been described elsewhere [18]. Haemoglobin (g/dl) was measured with a haemoglobinometer (Accuscience, Ireland) from a finger prick blood sample. The haemoglobinometer was calibrated daily prior to fieldwork. Auxillary temperature was taken with a digital thermometer. All children received 10 ml of oral multivitamins (over two days) as an incentive at each time point. Each 5 ml of multivitamin contained: Vitamin A 3000 IU, Vitamin B2 2.0 mg, Nicotinamide 15.0 mg, Vitamin B1 1.5 mg, Vitamin B6 2.0 mg, Vitamin D2 400 IU, D panthenol 1.0 mg.

### Statistical analysis

Statistical analysis was carried out in SPSS 14.01 and R v2.6.2 [25]. Socio-economic status (SES) was calculated as a score based on the number of key possessions (type of toilet facilities, generator, fridge, television, radio, mobile phone) in each child's household. A measure of household income was also included in the index calculation.

Children in the treatment and placebo groups were compared on the basis of their baseline characteristics: age, sex, village, SES, parasitic infections and clinical indicators (see Table 1). A similar analysis was also undertaken to compare the characteristics of children that were lost to follow-up and children that were included in the analyses. Children were included in the analyses if they complied with the four assessments and follow up at 14 months. Statistical analyses were carried out using Chi-squared tests for proportions (or Fisher's exact test when more than 20% of the cells had expected counts <5), two-sample t-tests to test eggs per gram (epg) and SES and Mann Whitney U tests to test continuous variables that were non normally distributed.

**Table 1:** Baseline characteristics - comparing individuals who did not fully comply and individuals who were analysed and individuals in the treatment and placebo groups

	Individuals who did not comply (N = 908)	Analysed individuals (N = 320)	P-value	Treatment Group (N = 158)	Placebo Group (N = 162)	P-value
**Characteristic**						
Age (years)						
1	314 (34.6%)	108 (33.8%)	0.137^a^	59 (37.3%)	49 (30.2%)	0.085^a^
2	202 (22.2%)	86 (26.9%)		38 (24.1%)	48 (29.6%)	
3	204 (22.5%)	76 (23.8%)		31 (19.6%)	45 (27.8%)	
4	188 (20.7%)	50 (15.6%)		30 (19%)	20 (12.3%)	
Sex						
Male	475 (52.3%)	159 (49.7)	0.419^a^	85 (53.8%)	74 (45.7%)	0.146^a^
Female	433 (47.7%)	161 (50.3)		73 (46.2%)	88 (54.3%)	
Village						
Akinlalu	112 (12.3%)	73 (22.8%)	< 0.001^a^	33 (20.9%)	40 (24.7%)	0.708^a^
Ipetumodu	407 (44.8%)	128 (40%)		65 (41.1%)	63 (38.9%)	
Moro	170 (18.7%)	49 (15.3%)		27 (17.1%)	22 (13.6%)	
Edun-abon	219 (24.1%)	70 (21.9%)		33 (20.9%)	37 (22.8%)	
Socio-economic status index						
Mean ± SE	6.52 ± 0.11	6.29 ± 0.06	0.058^b^	6.49 ± 0.15	6.56 ± 0.16	0.757^b^
**Parasitic infections**						
*Plasmodium *spp.^f^	-	-		71 (44.9%)	54 (33.3%)	0.033^a^
Mean parasitaemia^f^	-	-		2319 ± 511	1471 ± 341	0.025^b^
*Ascaris lumbricoides*	439 (48.3%)	146 (45.6%)	0.402^a^	73 (46.2%)	73 (45.1%)	0.838^a^
Mean epg ± SE	1080 ± 167	1075 ± 88	0.500^e^	1146 ± 283	1016 ± 183	0.926^b^
*Trichuris trichiura *^b^	36 (4%)	10 (3.1%)	0.496^a^	4 (2.5%)	6 (3.7%)	0.750^d^
Hookworm^b^	40 (4.4%)	13 (4.1%)	0.795^a^	4 (2.5%)	9 (5.6%)	0.171^a^
*Schistosoma haematobium *^b^	11 (1.2%)	3 (0.9%)	1^a^	0 (0%)	3 (1.9%)	0.248^d^
**Clinical indicator**						
Malaria attacks^f^	-	-		4 (2.5%)	4 (2.5%)	1^d^
Haemoglobin (g/dl; mean ± S.E.)	9.75 ± 0.05	9.69 ± 0.09	0.542^b^	9.54 ± 0.12	9.88 ± 0.11	0.039^b^
Weight (kg; mean ± S.E.)	11.98 ± 0.09	11.56 ± 0.14	0.019^b^	11.48 ± 0.20	11.64 ± 0.21	0.576^b^
Height (cm; mean ± S.E.)	88.01 ± 0.35	86.61 ± 0.51	0.064^e^	85.64 ± 0.87	87.02 ± 0.72	0.221^b^

^a ^χ^2 ^test

^b ^t-test

^c ^Mean epg not given as the number of eggs detected was very low

^d ^Fisher's exact test

^e ^Mann Whitney U

^f ^Data on malaria was only available for those individuals who were analysed

To account for the difference in Plasmodium prevalence and Plasmodium parasite density between the treatment and placebo groups at baseline, the analyses compared the rate of change in Plasmodium prevalence, Plasmodium parasite density and haemoglobin in the treatment group with the rate of change in the placebo groups. The Plasmodium prevalence data were analysed using a linear mixed effects model using a logit function with the assumption that the observed data were binomially distributed. The analyses were run in R v2.6.2 using the function *lmer *contained in the package *lme4*. A random effect for each individual was included to take account of the nested data structure (multiple observations made on each child). The effect of treatment (treatment or placebo), village and sex were included as fixed factors, and time as a linear covariate. Data were analyzed initially for all age groups combined (1-4 year olds at enrolment) and then identical analyses were performed on each separate age group (1, 2, 3 and 4 years). Parameter effects in the linear mixed effects models are estimated on a linear scale as log(*odds*), their effects are correspondingly additive with respect to their intercept, and the presented standard errors of the estimates are symmetrical. In order to determine the proportional change in *odds *referred to in the text, we calculate exp(log(*odds*)) so that the effects on the odds scale are correspondingly multiplicative with respect to their intercept.

*Plasmodium *parasite density was measured at baseline, 4, 8, 12 and 14 months. *Plasmodium *parasite density at baseline was subtracted from the densities at 4, 8, 12 and 14 months to provide a measurement for the rate of change in malaria parasite density. Since the data were not independent it was analysed by repeated measures rmANOVA (General Linear Model) with the different time points as a within-subject factor. Group, village, age and sex were chosen as the between-subject factors. The distribution of *Plasmodium *parasite density was not normal and therefore was log-transformed for the purposes of statistical analysis. The malaria attack data was not analyzed because the data were sparse and over-dispersed.

Haemoglobin concentration was measured at 0, 4, 8, 12 and 14 months from the same individuals and was analysed using rmANOVA using the same procedure as for the *Plasmodium *parasite density. The haemoglobin measurement at baseline was subtracted from the measurement at 4, 8, 12 and 14 months to provide a measurement for the change in haemoglobin (g/dl) from baseline. This was done on an individual subject basis.

The drug used to treat malaria infection was Coartem (artemether-lumefantrine). The difference in the total intake of Coartem, prescribed by the study doctor, between the treatment and placebo group was tested using a chi-squared analysis.

## Results

### Baseline characteristics

**Figure 1:**
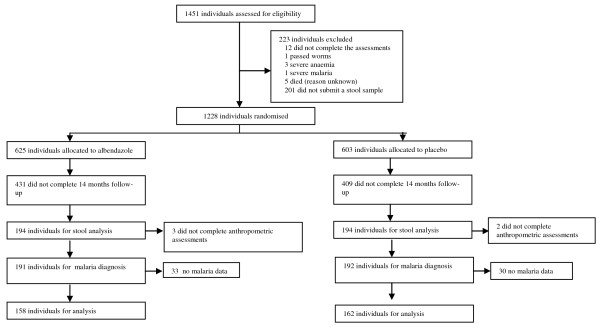
**Trial Profile**.

Of the 1228 children, 320 (26.1%) complied with all the follow-up assessments. Figure 1 shows the trial profile. The baseline characteristics were similar for those children who completed the trail and those who were lost to follow-up (Table 1), with two exceptions as follows: (i) fewer children from Akinlalu village were lost to follow-up than in the other three villages and (ii) children that were lost to follow-up had a significantly higher mean weight than children who completed the trial. Therefore the randomized design resulted in a similar distribution of most of the general physiological baseline variables between the treatment and placebo groups (Table 1).

At the time of recruitment into the study, there was a significantly higher prevalence of *Plasmodium *and greater *Plasmodium *density in the treatment group when compared to the placebo group, although the infection status of the children was not known when they were allocated into the treatment groups. In the linear mixed effects model analysis children in the treatment group had approximately twice the odds of having malaria than children in the placebo group (effect of treatment, P = 0.001, Table 2). Correspondingly, mean haemoglobin concentration was also significantly lower in the treatment group at baseline when compared to the placebo group.

**Table 2:** Linear mixed effects model for the prevalence of *Plasmodium spp*. in children of all ages, with parameter estimates (expressed as both odds and log-odds) and associated standard error of the estimate (of the log-odds coefficients) and p-values.

Coefficient	Odds	Log Odds Estimate	Std. Error	P-value
Intercept	0.46	-0.79	0.25	0.002
Group(treatment)	2.10	0.72	0.22	0.001
Akinlalu	1	0	-	-
Ipetumodu	0.47	-0.75	0.20	< 0.001
Moro	1.10	0.05	0.25	0.83
Edunabon	0.58	-0.54	0.22	0.01
Age	1.18	0.16	0.07	0.02
Time	1.15	0.14	0.14	< 0.001
Time:Group(treatment)	0.94	-0.07	0.02	0.002

AIC value = 2027

### Effect of anthelminthic treatment on *Ascaris *infection

The most prevalent STH amongst the children was *A. lumbricoides *(Table 1). The effect of four-monthly anthelmintic treatment on *Ascaris *infection has been described in a previous publication [18]. However, for clarity, a brief description is included here. The study demonstrated that repeated four-monthly anthelminthic treatment was successful in reducing prevalence and intensity of *A. lumbricoides *infections in preschool children. At the end of the follow-up period, 12% and 43% of the children were infected with *A. lumbricoides *and mean epg was 117 (S.E. 50) and 1740 (S.E. 291) in the treatment and placebo groups respectively compared to 45% and 45% of the children infected with *Ascaris *and mean epg being 1095 (S.E. 237) and 1126 (S.E. 182) in the treatment and placebo respectively at baseline. Of importance was the observation, that it took three rounds of anthelmintic treatment before the prevalence of *Ascaris *dropped in the treatment group. In contrast, intensity of ascariasis dropped after one round of anthelmintic treatment [18].

### Effect of anthelminthic treatment on *Plasmodium *infection

**Figure 2:**
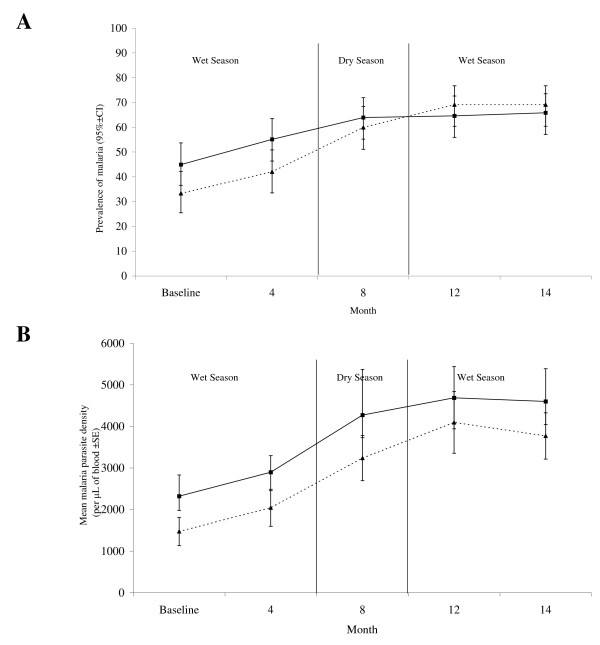
**(A) Prevalence rates (*Plasmodium spp*.) and (B) mean malaria parasite density in treatment (solid line) and placebo (dotted line) groups during the follow-up period**. Treatment group n = 158, Placebo group n = 162.

During the study period, the prevalence of *Plasmodium *infection had increased for the first 8 months of the study for children in the treatment group and for the first 12 months for children in the placebo group (Figure 2A). The steepest increase in the prevalence of *Plasmodium *infection in the placebo group from 8 to 12 months occurred from the dry to the wet season while the prevalence of *Plasmodium *infection in the treatment group plateaued. By 14 months, the prevalence of *Plasmodium *infection had increased by 46.5% and 107% in the treatment and placebo groups respectively when compared to the prevalence at baseline (Figure 2A). Infection reached a plateau for both groups (65% in the treatment group and 69% in the placebo group) by 12-14 months after the start of the study.

**Figure 3:**
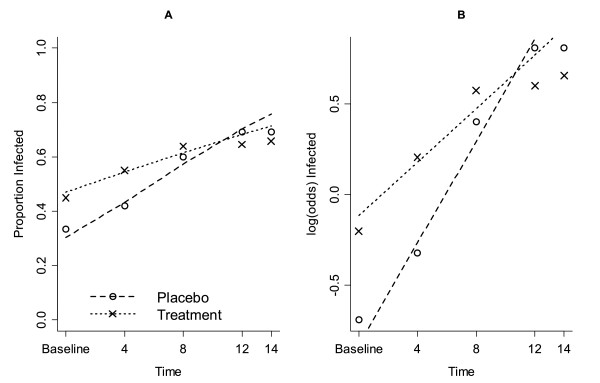
**(A) The proportion of individuals with *Plasmodium *spp**. parasites and (B) the log odds of individuals being infected with *Plasmodium *spp. parasites over time.

The odds of having *Plasmodium *infection increased over time for children in the placebo group, increasing proportionally by 1.15 per month (Figure 3AB; Table 2, effect of time P < 0.001). Children in the treatment group also increased in their odds of having *Plasmodium *infection over time (Figure 3AB), however this increase was significantly slower than the placebo group (Table 2, interaction between time and treatment, P = 0.002) and their odds increased by 1.08 per month (Table 2, 1.15 × 0.94 = 1.08). A similar analysis was carried out separately for each age group and all age groups in the treatment group showed a slower increase in *Plasmodium *infection although this effect did not reach statistical significance at 95% confidence level (data not shown).

*Plasmodium *density increased from baseline reaching a plateau at 12 months in both the treatment and placebo groups (Figure 2B). By 14 months, mean *Plasmodium *density increased by 156% (mean *Plasmodium *density at baseline of 1470 parasites per μL of blood to 3768 at 14 months) in the placebo group and by 98% (mean *Plasmodium *density at baseline of 2319 to 4601 at 14 months) in the treatment group. The treatment and placebo groups showed the same pattern in the rate of change in *Plasmodium *density throughout the study period (rmANOVA, interaction time*group (within subject analysis) F_2, 558 _= 0.456, P = 0.628). The rate of change in *Plasmodium *density did not significantly differ between the treatment and placebo groups (main effect of group (between subject analysis) F_1, 288 _= 0.025, P = 0.874).

The prevalence of clinical malaria attacks was low throughout the study period (Table 1) ranging from 3% at 4 months to 7% at 14 months. There was no significant difference in the intake of Coartem between treatment (50, 31.6%) and placebo (48, 29.6%) groups.

### Effect of anthelminthic treatment on haemoglobin level

**Figure 4:**
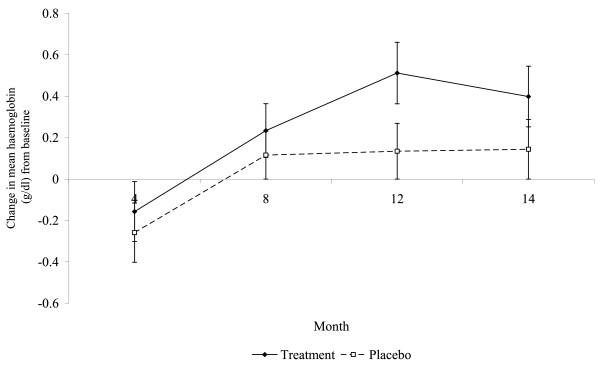
**Change in haemoglobin among the children in the treatment and placebo groups throughout the study period**.

Mean haemoglobin level remained higher in the placebo group compared to the treatment group throughout the study period, except at the 12 month survey point where the haemoglobin level was similar for both groups (10.04 g/dl ± S.E. 0.11 in the treatment group and 10.03 g/dl ± S.E. 0.11 in the placebo group; data not shown). Mean haemoglobin level increased by 4%, from 9.54 g/dl at baseline to 9.94 at 14 months, in the treatment group and increased by 2.3%, from 9.88 g/dl at baseline to 10.11 at 14 months, in the placebo group. The change in mean haemoglobin level varied over time, increasing from -0.209 at 4 months to 0.313 at 14 months (Table 3; rmANOVA, main effect of time (within subject analysis) F_3, 843 _= 6.714, P < 0.001). The change in haemoglobin levels increased from 4 months to 8 months in both the treatment and placebo groups and while the change stabilized in the placebo group it increased in the treatment group to 12 months and decreased slightly thereafter (Figure 4). Although the pattern in the change of haemoglobin concentration seemed to differ between the treatment and placebo groups this was not significant (Table 3; interaction time*group (within subject analysis) F_3, 843 _= 0.893, P = 0.445). There was no difference in the change in haemoglobin concentration between the treatment and placebo groups (Table 3; main effect of group (between subjects analysis) F_1, 281 _= 1.981, P = 0.160). The pattern in the change of haemoglobin concentration significantly differed between the villages over time (Table 3; interaction time*village (within subject analysis) F_3, 843 _= 3.128, P = 0.001). Change in haemoglobin concentration increased from 4 to 12 months in Ipetumodu and Edunabon in both the treatment and placebo groups and decreased thereafter. The pattern of change in haemoglobin was different in Akinlalu and Moro. In Akinlalu change in haemoglobin concentration in both the treatment and placebo groups increased from 4 to 8 months, decreased from 8 to 12 months and increased thereafter (data not shown). Change in haemoglobin concentration in Moro, in both the treatment and placebo groups, decreased from 4 to 8 months, increased from 8 to 12 months and decreased thereafter (data not shown). There was no difference in the change in haemoglobin concentration between the villages (Table 3; main effect of village (between subjects analysis) F_1, 281 _= 0.156, P = 0.926).

**Table 3:** Test of within- and between- subject effects from rmANOVA analysis on the change in haemoglobin concentration from baseline in treatment and placebo groups over the study period.

Source	Degrees of Freedom	F	P-value
**Within-Subject Effects**			
Time	3	6.714	P < 0.001
Time*group	3	0.893	0.445
Time*village	9	3.128	0.001
Time*age	9	0.728	0.684
			
**Between-Subject Effects**			
Group	1	1.981	0.160
Village	3	0.156	0.926
Age	3	2.189	0.089
Error	281		

## Discussion

Repeated four-monthly anthelminthic treatments for 14 months resulted in a significantly lower increase in the prevalence of *Plasmodium *infection in children aged 1 to 4 years which coincided with a reduction in both the prevalence and intensity of *A. lumbricoides *infections. This suggests that when children of this age group become infected with *Plasmodium*, infection with *Ascaris *could potentially influence the ability of the child's immune response to clear the *Plasmodium *infection. The immunological mechanism of action that underlines helminth/malaria interactions has been discussed and some authors suggest that helminths can contribute to the slow acquisition of immunity to malaria [26].

The greater increase in *Plasmodium *parasite density in the placebo group further supports the evidence that suggests that *Ascaris *can leave children aged 1 to 4 years less able to control *Plasmodium *infection although this was not statistically significant. This potential positive impact of anthelminthic treatment on *Plasmodium *infections in pre-school children contrasts with the results of other studies investigating the relationship between *A. lumbricoides *and malaria [5,6,8]. Two studies using an intervention have been undertaken in the Comoros islands [6] and Madagascar [5]. Both studies have included subjects with a wide age range, 2-14 yrs and 0 - >15 yrs, respectively. In the Comoros study, children with heavy *Ascaris *infections demonstrated an increase in clinical malaria cases with anthelmintic treatment. In the present study, the significant effect of anthelmintic treatment on *Plasmodium *infections was demonstrated in a group of children where the majority (85.9%) had light *Ascaris *infections [18]. The Comoro study had limitations and has been criticized for its small sample size (112), short follow-up (20 days) and severely malnourished study population [17].

In Madagascar, Brutus and colleagues revealed that while there was no effect of levamisole treatment on *P. falciparum *parasite density in children aged < 5 years, subjects more than five years of age had a significant increase in their *P. falciparum *parasitaemia compared with untreated controls[5]. The small sample size (n = 67) in the six month to four year age group may not have been sufficient to detect an effect of anthelmintic treatment on *P. falciparum *parasitaemia. Furthermore, the prevalence of *A. lumbricoides *in this age group may have been very low in comparison to these Nigerian children considering that the prevalence in the treatment (26.2%) and placebo (27.4%) groups was moderate at entry into the study. Differences in acquired malaria immunity, owing to different transmission settings, in the Nigerian and Madagascan population may also have contributed to the contrasting results.

An observational field-based, case-control study in rural Senegal investigated the relationship between *A. lumbricoides *and severe malaria [27] demonstrating that children infected with *A. lumbricoides *had an increased risk of severe malaria. This is in line with the results of the present study which demonstrate that children infected with *A. lumbricoides *are apparently less able to control their malaria infections, albeit uncomplicated malaria infections. Other studies investigating the relationship between helminths and malaria, not differentiating between helminth species, have also shown comparable results to this study [11,28].

In the present study, as expected haemoglobin levels increased over the study period as the children increased in age [29]. However, There was a steeper increase in the change in haemoglobin level among children in the treatment group when compared to the placebo group. This trend corresponds with the lower increase in prevalence of malaria infections associated with the transmission season in the treatment group. Malaria infection is associated with a reduction in haemoglobin levels and can frequently lead to anaemia[30], especially in younger children and infants [31]. Despite the fact that the trend in haemoglobin level was not statistically significant, the observations further support the evidence that *Ascaris *can leave these preschool children less able to control their *Plasmodium *infections.

The study may have some potential limitations that need to be considered when interpreting the findings. *Plasmodium *prevalence and parasite densities were significantly lower in the placebo group at baseline and thus it could be argued that that the placebo group had more potential for increasing *Plasmodium *prevalence and parasite densities than the treatment group. However, *Plasmodium *prevalence was comparable in the treatment and placebo groups at 8 months and while prevalence increased in the placebo group from the dry to the wet season, when malaria transmission was likely to be low, the prevalence reached a plateau in the treatment group. This trend does not support the theory that the faster increase in *Plasmodium *prevalence in the placebo group was due to differences in *Plasmodium *prevalence at baseline. Participants were not randomly selected from the community. Achieving a random sample of study participants in this field setting would prove very difficult owing to the widely dispersed nature of these semi-urban communities, and to the restricted age group being studied. Nevertheless, we believe that the moderate sample size, small age range and randomised design may compensate for the non-random selection of study participants. The majority of children (74%) were lost to follow-up. This high attrition rate may be attributed to the difficulty in retaining the participation of such young children particularly because they are not of school-going age. School-based studies provide a better infrastructure and can improve compliance considerably[32]. Bias caused by such losses was probably minor because those lost were similar with respect to important characteristics to those that were analyzed, apart from village and weight. Akinlalu is less widely dispersed than the other villages and this may account for the higher compliance shown in Akinlalu and any potential bias owing to this was taken into account by adjusting for village in the analyses. We do not believe that the intake of Coartem had any effect on the results because few treatments (98) were given during the study period and there was no significant difference in the intake of Coartem between the two groups. Information on whether the children received treatment for malaria prior to the assessments was not recorded. The inhabitants of these semi-urban villages have poor access to healthcare. The Primary Health Care centres have inadequate medical supplies and the staff are often poorly trained. Although malaria is hyperendemic in Osun state, the authors believe that the children did not receive appropriate treatment for malaria outside of the study setting.

## Conclusions

This study has demonstrated that the odds of having *Plasmodium *infection increased over time for children who received both treatment and placebo, however this increase was significantly slower in the treatment group. This observation therefore provides some evidence to suggest that infection with *A. lumbricoides *leaves these preschool children less able to control their *Plasmodium *infection.

Previous research has shown that repeated two-monthly anthelminthic treatments can have a significant impact on *Plasmodium *infection [5] and the present study suggests that longer intervals of treatment can also have a beneficial effect. The four-monthly treatments used here are also a better representation of the delivery frequency of anthelminthics in deworming control programmes [33]. The potential impact of anthelminthic treatment on *Plasmodium *infections demonstrated in these preschool children implies that large-scale deworming programmes may have a protective effect on malaria morbidity in these children in this particular setting.

Recently, the potential opportunities for combined control of malaria and helminths have been recognised[34]. Efforts are already underway in central Nigeria where the distribution of insecticide-treated bed nets has been combined with mass drug administration for lymphatic filariasis and STHs[35]. Treating children and adults concurrently for helminth and *Plasmodium *infections may be prudent as interactions may vary with helminth species which is plausible as different helminths and protozoa have synergistic and antagonistic interactions[36]. The integrated management of *Plasmodium *and helminth infection could avoid possible unforseen effects of mass distribution of anthelminthic drugs on malaria. There is still much we do not know about helminth-malaria interactions. While the results of this study provide some illumination of potential interactions between *A. lumbricoides *and malaria in children aged 1-4 years, we do not know what the outcome of these interactions will be in populations from different geographical settings in areas of low malaria transmission. It is crucial that this topic receives more attention, in light of a renewed emphasis on deworming and the fact that integrated control programs for malaria and helminths are not yet widespread.

## Competing interests

GlaxoSmithKline sponsored the drug albendazole which was used in the study. The authors declare that they have no competing interests. The authors also declare that they have no financial competing interests.

## Authors' contributions

CVH conceived the study and designed it with PK with input from all the other authors. SOA and TCA chose the villages and provided input for the logistics of fieldwork. PK conducted the fieldwork with SM and contributions from the other authors. PK did the stool analysis, carried out statistical analysis with ALJ and input from CVH, and drafted the manuscript. All authors contributed to the final version of the manuscript and read and approved it.

## Pre-publication history

The pre-publication history for this paper can be accessed here:

http://www.biomedcentral.com/1471-2334/10/277/prepub
